# Fluoride-Induced Negative Differential Resistance in Nanopores: Experimental and Theoretical Characterization

**DOI:** 10.1021/acsami.1c18672

**Published:** 2021-11-04

**Authors:** Jose J. Perez-Grau, Patricio Ramirez, Vladimir Garcia-Morales, Javier Cervera, Saima Nasir, Mubarak Ali, Wolfgang Ensinger, Salvador Mafe

**Affiliations:** † Departament de Física Aplicada, Universitat Politècnica de València, E-46022 Valencia, Spain; ‡ Departament de Física de la Terra i Termodinàmica, Universitat de València, E-46100 Burjassot, Spain; § Department of Material- and Geo-Sciences, Materials Analysis, Technische Universität Darmstadt, Alarich-Weiss-Str. 02, D-64287 Darmstadt, Germany; ∥ Materials Research Department, GSI Helmholtzzentrum für Schwerionenforschung, Planckstrasse 1, D-64291 Darmstadt, Germany

**Keywords:** negative differential resistance, threshold voltage, synthetic nanopores, nanofluidic devices, alkali metal fluorides, memristive model

## Abstract

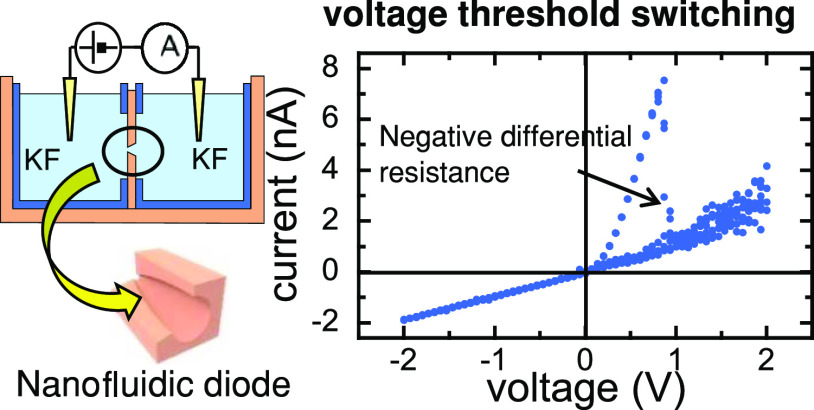

We describe experimentally and theoretically the fluoride-induced negative differential resistance (NDR) phenomena observed in conical nanopores operating in aqueous electrolyte solutions. The threshold voltage switching occurs around 1 V and leads to sharp current drops in the nA range with a peak-to-valley ratio close to 10. The experimental characterization of the NDR effect with single pore and multipore samples concern different pore radii, charge concentrations, scan rates, salt concentrations, solvents, and cations. The experimental fact that the effective radius of the pore tip zone is of the same order of magnitude as the Debye length for the low salt concentrations used here is suggestive of a mixed pore surface and bulk conduction regime. Thus, we propose a two-region conductance model where the mobile cations in the vicinity of the negative pore charges are responsible for the surface conductance, while the bulk solution conductance is assumed for the pore center region.

## Introduction

Counterintuitive negative differential resistance (NDR) phenomena occur when a sharp electrical current decrease follows an increase of the applied voltage beyond a threshold value. While this effect is well known in solid state electronic switches and memories, it remains mostly underexplored in liquid state ionic nanodevices. Recently, we have presented a preliminary account of the fluoride ion-induced NDR and threshold switching phenomena observed when the conical nanopores are bathed in KF solutions at a low concentration.^1^ Note here that other nanopore NDR effects previously reported are based on different experimental systems and physicochemical phenomena, including calcium-induced gating,^2^ mechanically forced electroosmotic flows,^3^ electrical field-modulated ionic transport,^4,5^ ionic-liquid/water mixtures,^6^ and pH-regulated mesopores.^7^

As a significant extension of our previous brief letter,^1^ we provide here a comprehensive experimental and theoretical description of the fluoride-induced NDR phenomena. To this end, we use both single pore and multipore membrane samples. Also, the experimental data concern different pore radii, charge concentrations, scan rates, salt concentrations, solvents, and cations. Under the NDR conditions, the threshold voltage switching occurs around 1 V and leads to sharp current drops in the nA range, with a peak-to-valley ratio close to 10. These facts suggest that small voltage changes around 1 V can amplify weak electrical perturbations, an effect potentially useful for nanofluidic applications in sensing and actuating.^8−10^

The effective radius of the pore tip zone is of the order of 10 nm, which is close to the Debye length of aqueous electrolyte solutions for concentrations in the range 1–10 mM, which is suggestive of a mixed conductance regime. Thus, we propose a two-region surface and pore center model where the mobile cations in the vicinity of the negative pore charges are responsible for the surface conductance while the pore central region is characterized by a bulk-like solution conductance.

Taking together, the experimental data and the model calculations show that ion-specific memory and memristor-like characteristics are significant here. Indeed, the dependence of the observed NDR effect on the anion and cation type, mobile ions concentration, scan rate, and pore effective radius suggests that the kinetically limited redistribution of ions and water molecules at the pore tip zone can be modulated by time-dependent perturbations. We believe that the external regulation of the nanopore NDR phenomena described here is of fundamental interest and may have sensing and actuating applications.

## Experimental

Single-pore and multipore polyimide (PI) and polyethylene terephthalate (PET) membranes were fabricated by foil irradiation with heavy ions. Subsequently, the ion tracks were converted into nanopores by exposing the polymer membrane to chemical etching with a strong inorganic etchant under asymmetric conditions.^11,12^ Different membrane samples were used to check the reproducibility and significance of the NDR phenomena. The membrane was placed in an electrochemical cell separating two salt solutions and no buffer solution was added. The solution pH was in the range 6.5–7.0 during the measurements. Because these pH values are higher than the p*K*_a_ characteristic of the −COOH moieties functionalized on the pore surface, the carboxylic acid groups were ionized to −COO^–^ and thus the pore was negatively charged.^13,14^

For the case of the positively charged pore, the membrane sample was immersed in an aqueous solution of polyethyleneimine (PEI, 5 mg/mL and pH ∼ 11) overnight.^15^ Under these conditions, the primary amine moieties of PEI chains were covalently linked with the carboxylate groups on the pore surface. The modified membrane was then exposed to an acidic aqueous solution (pH 3) to protonate the amine groups for 1 h. For this purpose, branched PEI having an average *M*_n_ ∼ 1200 with concentration of 50 wt % in H_2_O (Sigma-Aldrich) was used. The ion transport characteristics of the modified pores showed that the immobilized polymer chains could not affect the nanopore tip opening because of the relatively low molecular weight of PEI polymer.

The radii of the approximately conical pores were obtained by the imaging of the pore base and the measurement of the nanopore electrical conductance, for the case of the pore tip.^13,14,16,17^ The resulting pore radii were in the range 100–400 nm (base) and 10–40 nm (tip).^18,19^ We used also single pore samples with positive charges obtained after functionalization of the as-prepared pore with PEI chains.

Ag|AgCl electrodes incorporating 2 M KCl solution salt bridges were connected to a voltage-source picoammeter (Keithley Instruments, Cleveland, Ohio) for the electrical measurements. To check further that the NDR phenomena observed were due to the nanopore and independent of the electrode type used, Ag|AgCl electrodes without salt bridges and Pt electrodes were also used in separate control experiments. In order to isolate the electrochemical cell from environmental electrical perturbations, the experiments were conducted in a double-layered magnetic shield (Amuneal Manufacturing, Philadelphia, PA) placed on an antivibration table (Technical Manufacturing Corporation, Peabody, Massachusetts). The membrane was allowed to equilibrate with the appropriate 1–100 mM salt (KF, KCl, KBr, and KI; LiF, NaF, and KF) solution before each electrical measurement to assure data reproducibility.

## Results

**Figure 1:**
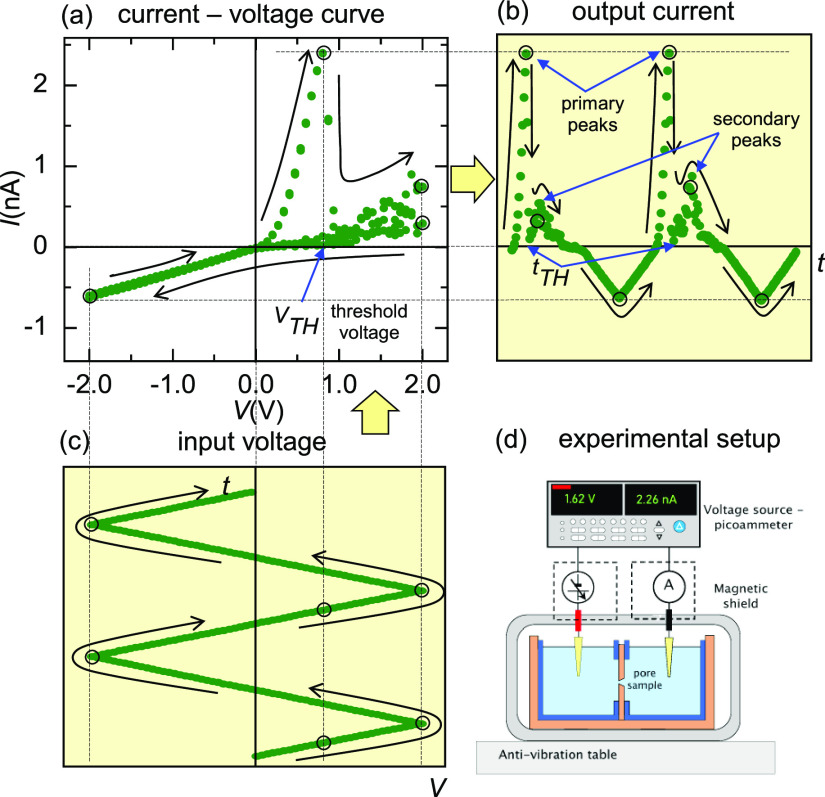
(a) *I*–*V* curve for a PI nanopore (sample # 1) in a 2 mM KF solution. The current rectification is due to the asymmetric charge distribution along the conical pore axis. (b) *I*–*t* curve is obtained by applying a triangular *V*–*t* signal (c). NDR effect is characterized by a sudden drop of the electric current at a threshold voltage *V*_TH_ ≈ 0.9 V for this pore. (d) Scheme of the experimental setup.

Figure 1a–d shows the NDR effect observed when a membrane containing a single asymmetric PI nanopore (sample # 1) separates two KF concentrations in the mM range. Because of the small currents measured, reproducible results were obtained after isolating the electrochemical cell in a double-shielded Faraday cage placed on an anti-vibration table (Figure 1d). The NDR region in the current–voltage (*I*–*V*) curve (Figure 1a) can be clearly seen as a sharp current drop (Figure 1b) when the input voltage (Figure 1c) exceeds a certain threshold value *V*_TH_ > 0. Triangular voltage versus time (*V*–*t*) input signals of amplitude 2 V are used to obtain the electrical readout of the pore. Between two subsequent measurements, the voltage was increased in Δ*V* = 67 mV, resulting in a scan rate of *ca.* 95 mV/s.

The primary peaks observed in the *I*–*t* curves for *I* > 0 correspond to those times *t*_TH_ where *V*_TH_ is attained. Under these conditions, *V*_TH_ is around 1 V for 1–10 mM fluoride solution concentrations, while the measured currents are in the nA range. After *V*_TH_ is exceeded, the current increases again with the input voltage, showing a noisy quasi-Ohmic behavior. At *V* = 2 V, a secondary peak is attained and, when the voltage begins to decrease, the current decreases accordingly with a significant noise reduction. No NDR effect is observed for *V* < 0.

**Figure 2:**
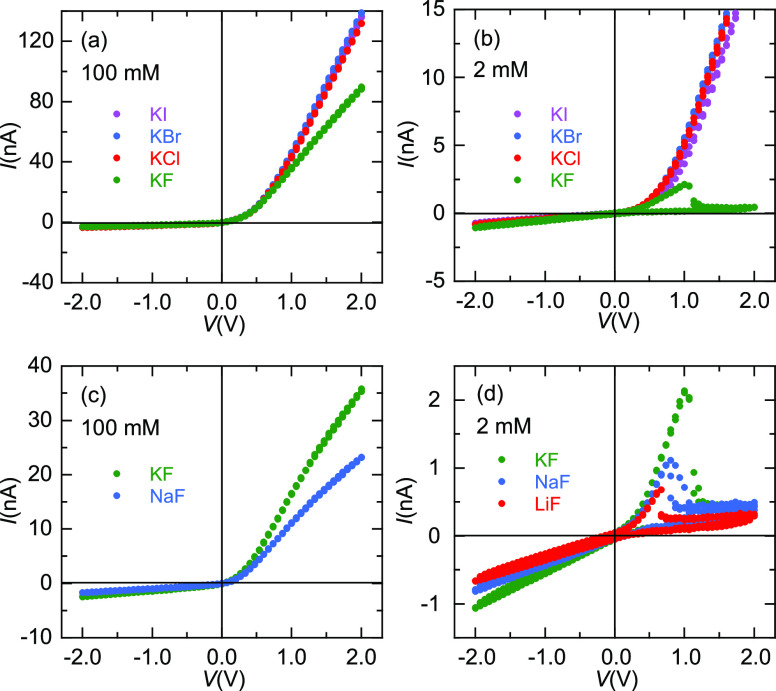
(a) *I*–*V* curves for KI, KBr, KCl, and KF at high (100 mM) and (b) low (2 mM) concentrations obtained with a PI nanopore (sample # 2). (c) *I*–*V* curves for NaF and KF at 100 mM and (d) LiF, NaF, and KF at 2 mM obtained with a PI nanopore (sample # 3). Note that LiF is not soluble in water at 100 mM. Clearly, the NDR phenomena observed are robust and appear to be a distinctive feature of the presence of F^–^ ions in the low concentration (mM) range.

The *I*–*V* curves of Figure 2a,b obtained for different potassium halides at 100 mM (Figure 2a) and 2 mM (Figure 2b) suggest that the NDR effect is a distinctive feature of F^–^ ions at concentrations in the mM range. The currents measured with KCl, KBr, and KI in Figure 2a are similar while those obtained with KF show significantly lower values, in good agreement with the dilute solution diffusion coefficient series *D*_Cl^–^_ = 2.03 ≈ *D*_I^–^_ = 2.05 ≈ *D*_Br^–^_ = 2.08 > *D*_F^–^_ = 1.48 in 10^–9^ m^2^/s units. In the low concentration range, however, only KF displays NDR (Figure 2b) while the other halides again show almost indistinguishable *I*–*V* curves.

The curves of Figure 2c,d were measured with NaF and KF at 100 mM (Figure 2c) and LiF, NaF, and KF at 2 mM (Figure 2d). In the curves of Figure 2c, no NDR is observed while in those of Figure 2d, the three alkaline fluorides show NDR at low salt concentrations. These facts suggest that specific effects due to the counterion-dominated pore swelling are not responsible for the NDR effect clearly observed and characterized here. Note also that the measured currents are in agreement with the dilute solution diffusion coefficient series *D*_Li^+^_ = 1.03 < *D*_Na^+^_ = 1.33 < *D*_K^+^_ = 1.96 in 10^–9^ m^2^/s units. The threshold voltage increases slightly following the sequence *V*_TH_ (LiF) < *V*_TH_ (NaF) < *V*_TH_ (KF), probably reflecting the different hydration energies of the cations.

**Figure 3:**
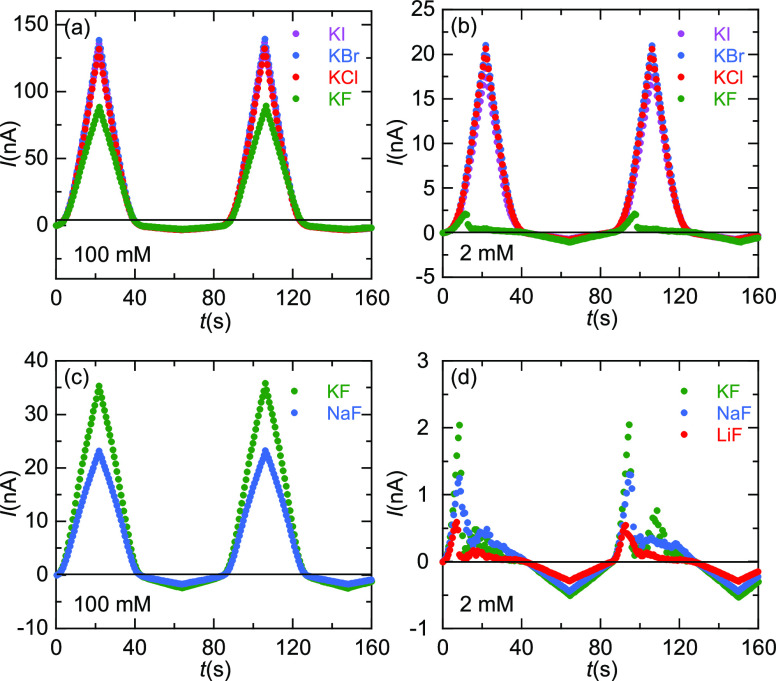
(a–d) *I*–*t* traces corresponding to the *I*–*V* curves of Figure 2.

To characterize further the observed phenomena, Figure 3 shows the *I*–*t* traces corresponding to the experiments of Figure 2. The curves of Figure 3a (high concentration, 100 mM) for KCl, KBr, and KI show high rectification ratios, defined as *r*_e_ = |*I*(*V*)/*I*(−*V*)|, with *r*_e_ ≈ 45 for *V* = 2 V. This value decreases to *r*_e_ ≈ 30 in the case of the 100 mM KF solution due to the relatively low pore conductance observed at *V* > 0 (Figure 2a). Note also that there is no time shift between the maximum values attained by the current for the different salts.

In the case of Figure 3b (low concentration, 2 mM) for KCl, KBr, and KI, we obtain *r*_e_ ≈ 25 at *V* = 2 V while for the KF salt showing NDR, we obtain *r*_e_ ≈ 0.4, thus reversing the pore rectification characteristics at this voltage. In Figure 3c (high concentration, 100 mM), the *I*–*t* curves show rectification ratios *r*_e_ ≈ 15 (KF) and *r*_e_ ≈ 13 (NaF) at *V* = 2 V, again with no time shift between the maximum current values. However, the three curves of Figure 3d (low concentration, 2 mM) for KF, NaF, and LiF show again NDR and reverse pore rectification characteristics, with *r*_e_ ≈ 0.4 at *V* = 2 V for the three fluoride salts. The time at which the NDR effect occurs follows also the sequence *t*_TH_ (LiF) < *t*_TH_ (NaF) < *t*_TH_ (KF).

**Figure 4:**
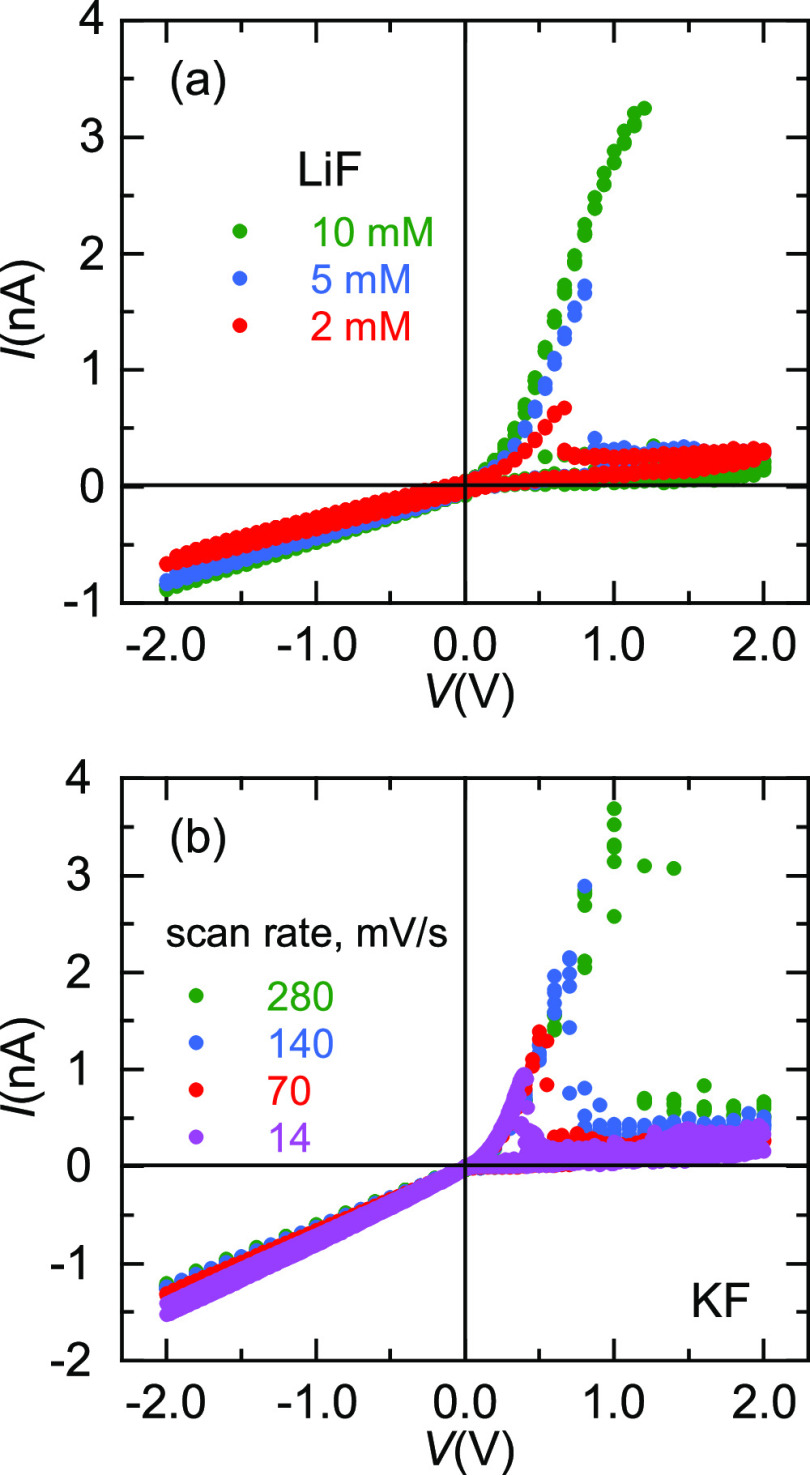
(a) *I*–*V* curves obtained with a PI nanopore (sample # 3) at the LiF concentrations shown in the inset. (b) *I*–*V* curves obtained with sample # 4 at the scan rates shown in the inset.

Figures 1–3 suggest that the NDR phenomena depend on the particular low concentration of the F^–^ ion and the time change of the input signal. Figure 4a shows the *I*–*V* curves of LiF at concentrations in the mM range. The threshold voltage *V*_TH_ increases with the salt concentration and vanishes at values higher than 20 mM LiF for this membrane sample (not shown). Previous experiments^1^ showed NDR effects up to 100 mM in the case of the KF salt and PI pores.

Figure 4b shows that the NDR depends on the scan rate of the signal. At low scanning rates, the nanopore has well-defined *V*_TH_ values and relatively low noise levels. Increasing the scan rate above 280 mV/s leads to a blurring of the NDR effect, driving the pore to the high conductance level for *V* > 0 and enhancing the current noise for *V* > *V*_TH_.

**Figure 5:**
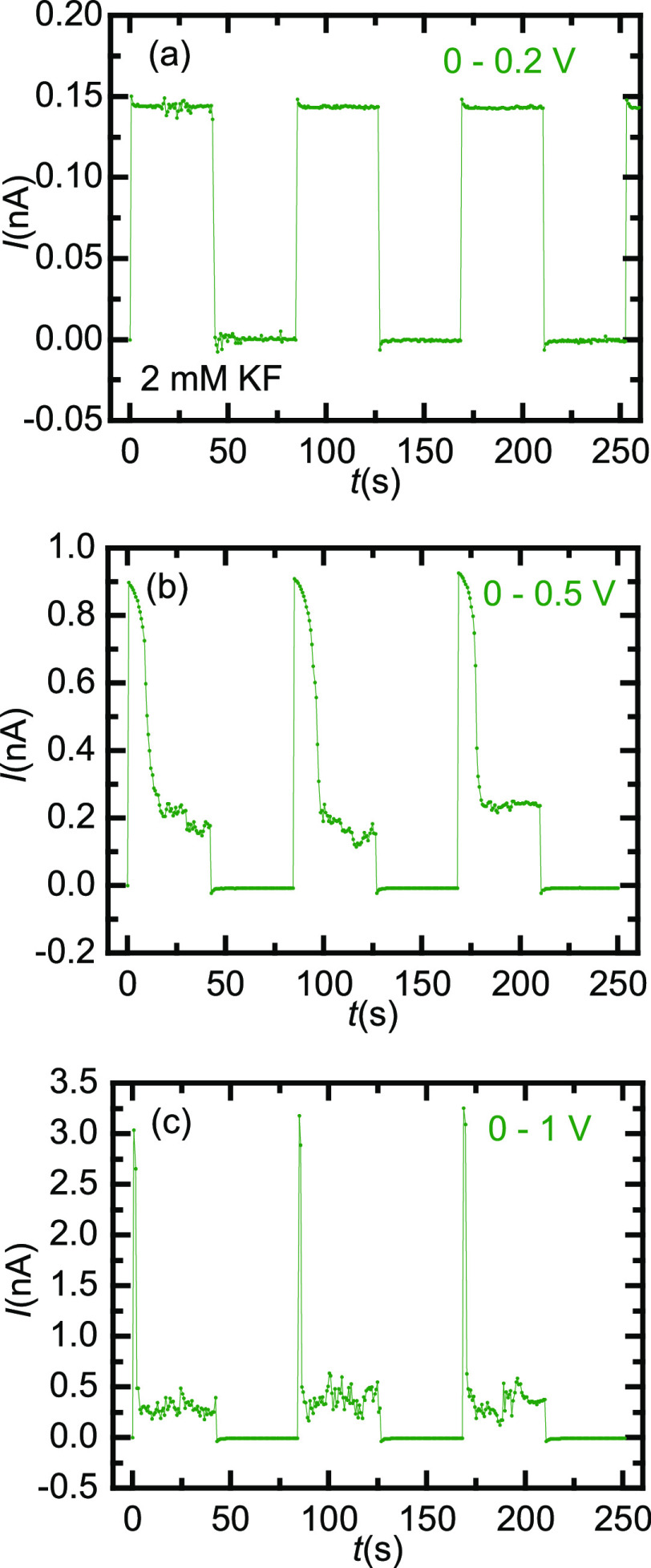
*I*–*t* traces obtained with a PI nanopore (sample # 1) and a rectangular *V*–*t* signal ranging (a) from 0 to 0.2, (b) 0 to 0.5, and (c) 0 to 1 V.

Figure 5 shows the pore response to rectangular input *V*–*t* signals ranging from 0 to 0.2 V (Figure 5a), 0.5 V (Figure 5b), and 1 V (Figure 5c). The salt concentration was 2 mM KF. The *I*–*t* traces of Figure 5a suggest that when the maximum voltage is lower than *V*_TH_, the NDR phenomenon has not been yet developed and the pore responds with a single current level. When the voltage bias is increased to values close to *V*_TH_ (Figure 5b), the pore responds first with a relatively high current as a result of the sharp increase of the voltage applied that drives the pore to the high conductance level. After a transient time, however, the pore relaxes to the low conductance level with a noisy steady current. For voltage bias above *V*_TH_ (Figure 5c), the NDR effect is fully developed, with small transition times between the two conductance regimes and high noise levels in the current. The time responses shown in Figure 5a–c demonstrate again the reproducibility and robustness of the phenomena characterized here.

**Figure 6:**
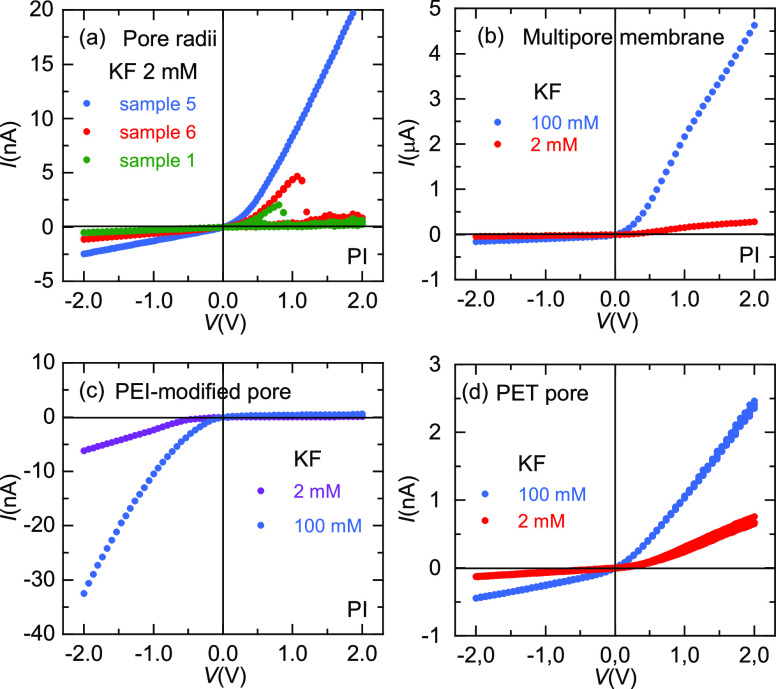
(a) *I*–*V* curves for three PI single pore membranes with different radii and a KF 2 mM solution. (b) *I*–*V* curves for a PI multipore membrane (5 × 10^3^/cm^2^ pores, sample # 7) at low and high KF concentrations. (c) *I*–*V* curves for a PEI modified PI single pore membrane (sample # 2). Note the opposite current rectification due to the positive rather than negative pore charge. (d) *I*–*V* curves for a PET single pore membrane (sample # 8).

As could be expected, the NDR effects are also influenced by the pore characteristics. Figure 6a shows the *I*–*V* curves obtained with 2 mM KF and three PI single pore samples whose characteristic parameters are shown in Table 1. These parameters have been estimated assuming pores with bullet-like tips and the PNP model described in refs (20) and (21). Here, *e* is the elementary charge and the parameter *d*/*h* describes the sharpness of the pore tip.^20^ According to our experience with the PI pores, much better agreements between the experimental and theoretical *I*–*V* curves are obtained by assuming a bullet-like rather than a perfectly conical pore geometry. In particular, this fact allows to explain the relatively high conductances and rectification rates observed in the PI pores compared with those of the PET pores where conical geometries are usually used.^21^ Note that *V*_TH_ increases with the pore radii so that the NDR phenomena are absent for the case of wide pore openings, which correspond to low pore charge concentrations at the region of the pore tip that controls the ionic transport characteristics.^14^ The NDR phenomena are also absent in the case of the PI multipore samples (Figure 6b) because a multitude of relatively wide pores dictate the ionic transport characteristics of the membrane in this case.

**Table 1:** Pore Parameters Estimated Assuming Bullet-like Tips and a PNP Model^20^ for the Samples Used in the Experiments of Figure 6a

sample	pore tip radius (nm)	pore base radius (nm)	surface charge concentration (*e*/nm^2^)	*d*/*h*
1	7	180	–0.8	18
5	100	450	–0.5	6
6	9	280	–0.6	14

The as-prepared PI samples containing negative surface charges can be converted into positively charged membranes after functionalization with PEI chains, as evidenced by the inverse rectification of Figure 6c obtained in 2 and 100 mM KF solutions. Note that before the PEI modification, the as-prepared sample showed NDR at 2 mM KF (Figure 2b). However, the modified pore (Figure 6c) does not show NDR in the concentration range examined. This fact gives further support to the assumption that it is F^–^ acting as a coion in the as-prepared pores that gives the NDR phenomena.

We have studied also a single pore PET membrane because these pores tend to show lower effective radii than those of the PI membranes, as evidenced by the lower conductances observed (Figure 6d). However, the PET membrane used do not show the NDR in the voltage and concentration ranges investigated. This fact suggests that additional factors such as the chemical nature of the different polymers of these two membranes, together with the smoothness of the etched surface,^16^ should impact on the membrane water content and the polymeric chains conformation, affecting thus the interaction between the F^–^ ions and the pore negative charges.

**Figure 7:**
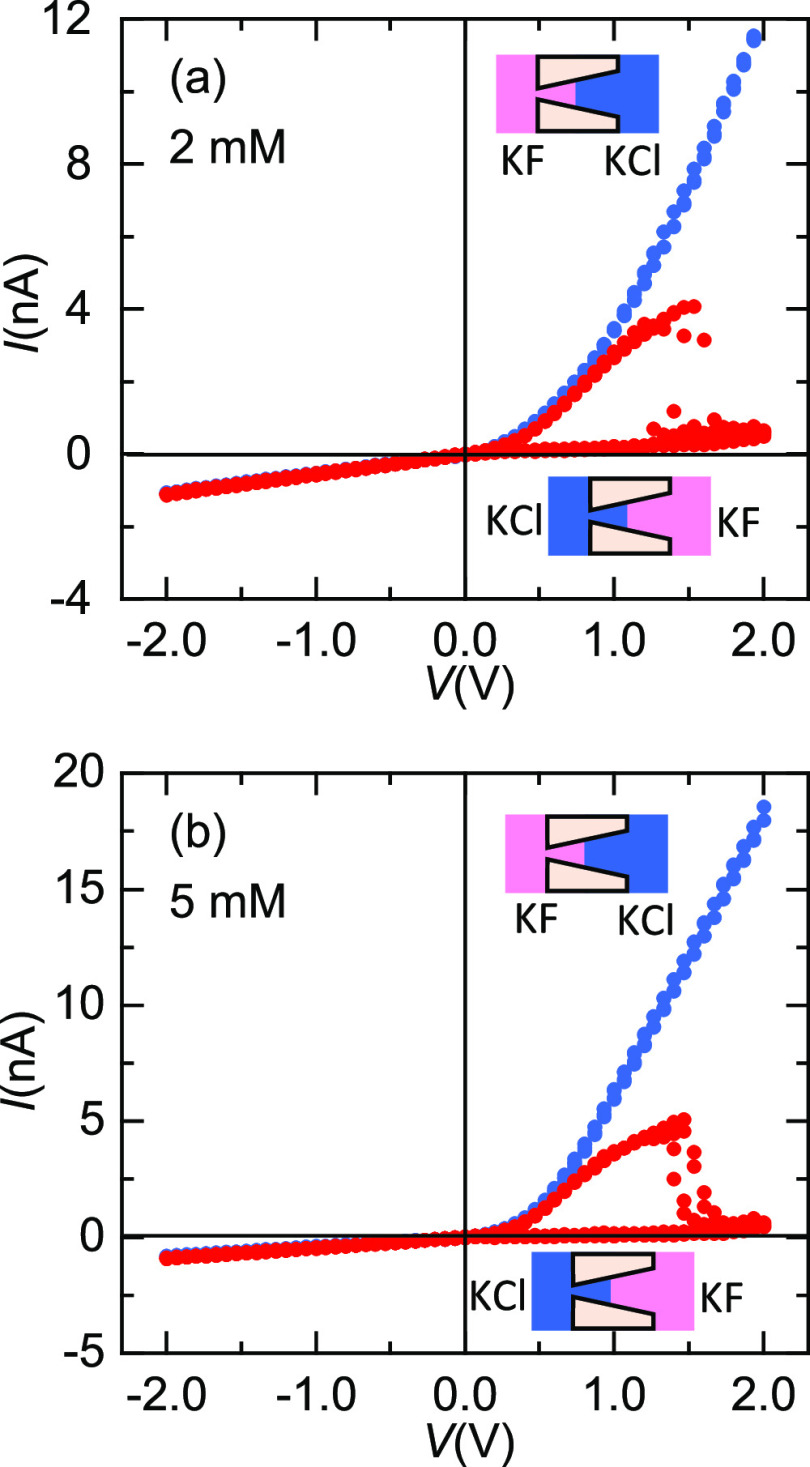
(a) *I*–*V* curves of a PI nanopore (sample # 4) separating 2 mM KF and KCl solutions at different orientations. (b) *I*–*V* curves of a PI single pore membrane (sample # 1) separating 5 mM KF and KCl solutions at different orientations. The insets indicate the relative position of the solutions with respect to the pore.

The presence of anions other than F^–^ in the external bathing solutions can also influence the NDR effect. Figure 7 shows the *I*–*V* curves of PI single pore membranes separating two different solutions at the same concentration, with KCl in one chamber and KF in the other chamber. Two different membranes and concentrations (2 mM in Figure 7a and 5 mM in Figure 7b) were considered under the experimental conditions indicated in the insets.

These results provide a new physical insight: NDR phenomena are only observed at *V* > 0 when the KF solution faces the pore wide opening and thus the F^–^ ions are driven by the imposed electric field from the base to the pore tip where they encounter the electrostatic barrier due to the negative pore tip charges.^14^ Indeed, no NDR effect is noted when the KCl solution faces the pore base so that the F^–^ ions are now driven from the tip to the pore base by this field. This fact confirms further the anionic-specific characteristic of the NDR effect, which is not observed with the Cl^–^ ions.

**Figure 8:**
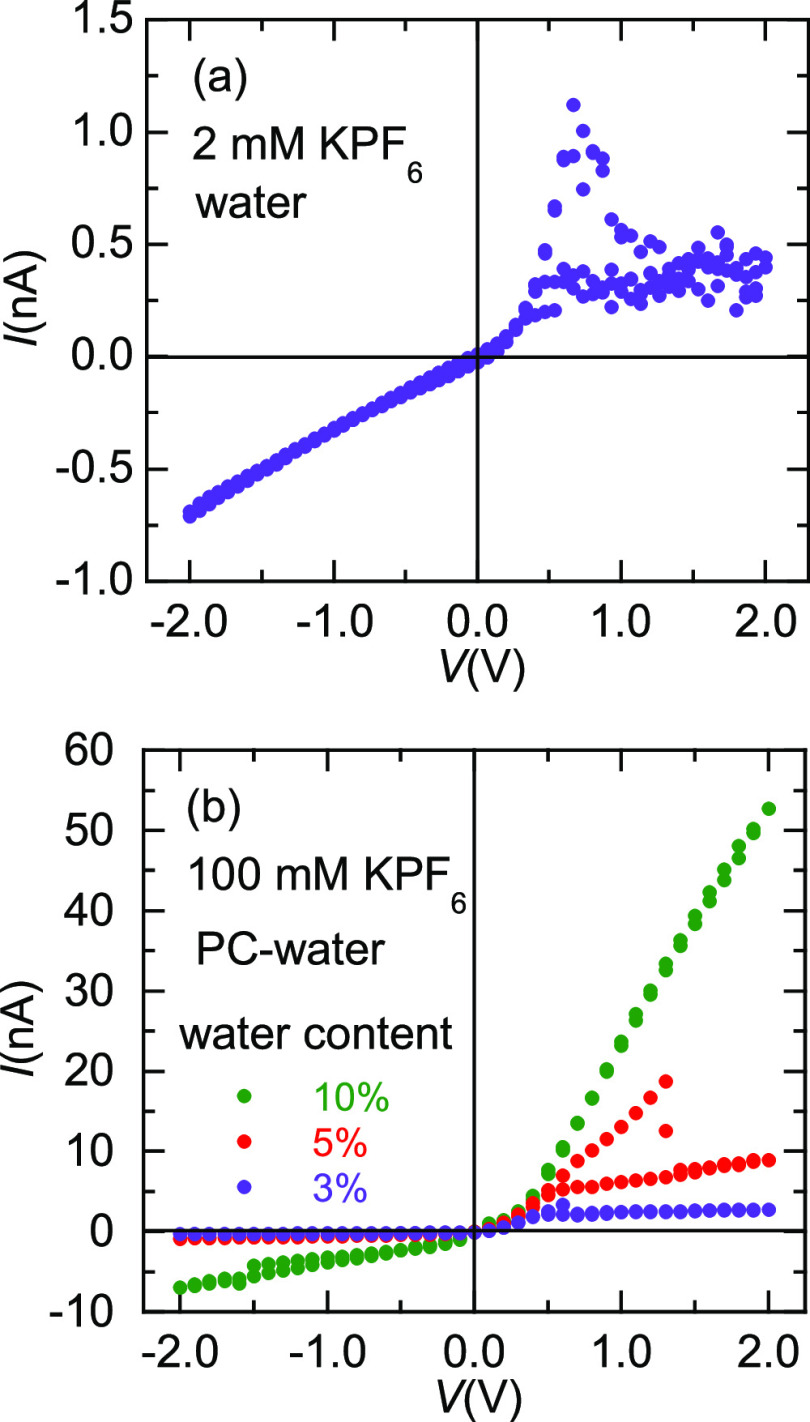
(a) *I*–*V* curve of a PI nanopore (sample # 9) separating 2 mM KPF_6_ solutions in water. (b) *I*–*V* curve of a PI single pore (sample # 10) separating 100 mM KPF_6_ solutions prepared in a mixture of PC and water with the water contents indicated in the inset.

We have also observed NDR phenomena in solutions containing other salts and solvents. Figure 8 shows the *I*–*V* curves of single nanopores separating two potassium hexafluorophosphate (KPF_6_) solutions. In the case of Figure 8a, the NDR effect appears at *V*_TH_ ≈ 0.6 V. In the curves of Figure 8b, NDR phenomena are observed for 3 and 5% water content, giving *V*_TH_ (3%) ≈ 0.6 V and *V*_TH_ (5%) ≈ 1.3 V. The NDR effect vanishes for water contents >7%. Interestingly, although the total concentration of KPF_6_ (100 mM) used in the curves of Figure 8b is higher than that of the aqueous solution of Figure 8a, the fact is that the NDR behavior is observed in the propylene carbonate (PC)–water mixtures only when the water dissolved PF_6_^–^ anion concentration is in the mM range.

**Figure 9:**
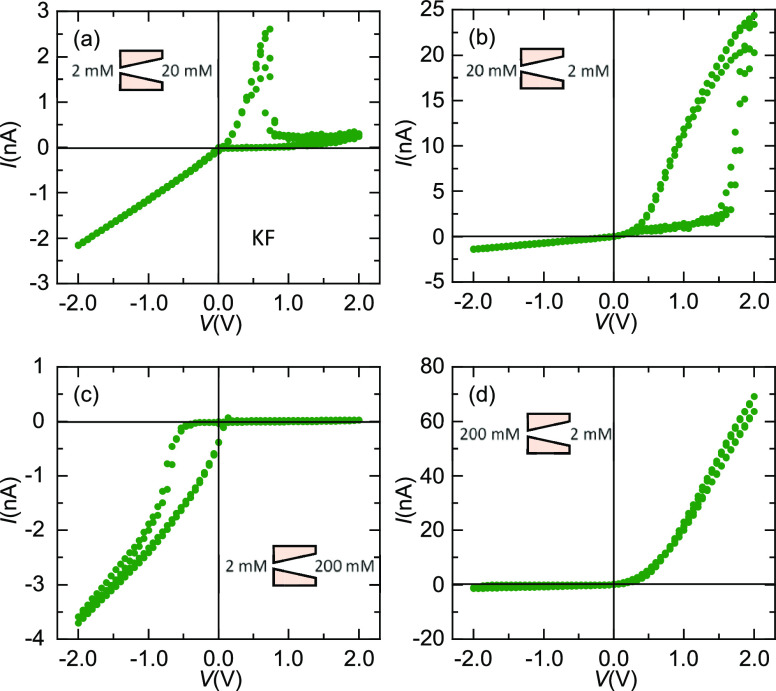
(a–d) *I*–*V* curve of PI nanopore (sample # 4) separating two KF solutions at the different concentrations and orientations indicated in the respective insets. Note the opposite current rectification due to the high concentration gradient across the pore combined with the low concentration value at the pore tip (c).

It is also interesting to consider single pores separating two KF solutions at different concentrations because saline gradients and asymmetric pores are usual in basic and applied studies. Figure 9 shows the *I*–*V* curves of a single pore PI membrane in the cases 2–20 mM KF (10-fold concentration gradient, Figure 9a,b) and 2–200 mM KF (100-fold concentration gradient, Figure 9c,d). The particular orientation of the salt gradient indicated in the insets shows again that the crucial role of the KF concentration at the pore tip region. When the 2 mM KF solution faces the pore tip (Figure 9a), a sharp current drop is obtained at *V*_TH_ ≈ 0.7 V, as observed in Figures 1, 2, 4, and 6 under analog experimental conditions. However, when the 20 mM solution is in contact with the pore tip (Figure 9b), the current drop becomes smoothed and *V*_TH_ increases to ca. 2 V, as observed when the KF concentration is increased.^1^ Note here that the F^–^ diffusion and conduction act in the same direction in Figure 9a, which results in a relatively low *V*_TH_ for the NDR effect to appear. On the contrary, F^–^ diffusion opposes to conduction in Figure 9b, which results now in a relatively high *V*_TH_ for the NDR effect to show up.

In Figure 9c,d (the case of the 100-fold KF concentration gradient), however, the pore behavior changes drastically. In Figure 9c, the concentration of the solution close to the pore tip is much lower than that close to the pore base. Thus, the pore shows higher resistance for *V* > 0 than for *V* < 0.^14^ Note also that, in this case, the 100-fold concentration gradient gives a K^+^ diffusion flux that acts against the electric field driven flux (ionic conduction), contrary to the case of Figure 9d where both potassium fluxes follow the same direction. As a result, the rectification characteristics of the pore are reversed with respect to those observed when there is no concentration gradient (Figure 2a) or when this gradient acts in the same direction as the electric potential gradient (Figure 9d).^4,22^

## Theoretical Model

We have presented previously a theoretical model^1^ based on a phenomenological memresistive approach.^23^ The model assumed two hypothetical pore resistances for the high and low conductances regimes attained following the *V* cycle. These presumed two pore states were connected by the NDR region observed when the voltage time cycle passes through the threshold voltage. While this model was useful, it is merely descriptive and does not provide clear physicochemical insights into the phenomena observed.^1^ We have proposed here a new theoretical scheme. Note the complexity of the problem that involves different factors such as the non-cylindrical pore geometry and the tip nanoscale, the chemical nature of the membrane polymer, and the interaction between the F^–^ ions, the alkali cations, the water molecules, and the negatively charged pore surface. Thus, we have resorted to a simple, tentative conceptual approach that could be elaborated further in future studies.

Experimentally, the fluoride ion has a relatively small ionic radius compared with the Cl^–^, Br^–^, and I^–^ anions. This ionic characteristic suggests a high surface charge density and hydration energy, with a strong immobilization of the surrounding water molecules, especially in confined nanoscale environments. In addition, the effective radius of the pore tip should be of the same order of magnitude than the Debye length (about 10 nm) for salt concentrations in the range 1–10 mM,^24^ which weakens the effective Debye screening of the surface pore charges. Under these conditions, bulk ionic conduction can be compromised at the narrow pore tip.^14^

Taking together, the above experimental facts are suggestive of a mixed pore surface and bulk conduction regime^25^ at *V* > 0, when the co-ions (F^–^ ions here) that accumulate at the pore tip barrier^14^ interact with the pore surface and compete for the water molecules needed for ionic conduction. Thus, as a complementary view to the experimental characterization of the NDR phenomena, we have developed a simple two-region conductance model that may allow for a qualitative description of the problem. In the pore surface region, the conductance is due to the mobile cations in the vicinity of the negative pore charges. In the pore center region, the conductance resembles that of the external electrolyte solution because a significant number of these pore charges are effectively neutralized by the cations in the surface region. Note that for the NDR effects to be significant, the contribution of the surface conductance to the total pore conductance should be of the same order of magnitude as the contribution of the pore center conductance.^25^

In the above theoretical approach, the fraction *f* = *X*/*X*_0_ (0 < *f* < 1) of the pore charges that gives the free cations available for surface conduction is assumed to be
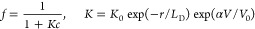
1only when the fluoride salts are used. In eq 1, *X*_0_ is the maximum volume concentration of pore charges,^25^*K*_0_ (mM^–1^) is the association constant between these charges and the cations, and *c* is the cation concentration in the external solution. Note that the pseudo-association constant *K* includes the Debye screening of the pore charges by the mobile ions at the pore center region, which is accounted for by the phenomenological factor exp(−*r*/*L*_D_) where *r* (nm) is the effective pore radius of the tip zone and  is the Debye length for a 1:1 salt in aqueous solution.^24^ In eq 1, the thermal potential *V*_0_ = *R*_g_*T*/*F* = 26 mV is written in terms of the gas constant *R*_g_, the temperature *T*, and the Faraday constant *F*.^24^

The voltage-dependent factor exp(α*V*/*V*_0_) of eq 1 accounts for a phenomenological voltage-assisted transference of the ions to the highly-charged pore tip from the adjacent zones. We assume here that when the fluoride ions enter the pore and perturb the pore tip hydration characteristics (voltage *V* > 0), it is the subsequent cation association with the negatively charged pore surface that is involved in the observed conductance changes. Because we ignore the microscopic details of this process, we assume that it is activated by the voltage drop α*V* at the tip, where α (0 < α < 1) is the dimensionless “electrical distance” that parametrizes this drop. This simplified approach is usually introduced in ion channel models.^26^ The effective voltage drop α*V* can be much higher than the typical equilibrium Donnan potentials, which are of the order of 10–50 mV only.^14,24^ Note also that the electric field associated with this voltage drop drives the cation and fluoride ion to the pore tip following opposite directions (Figure 1).

For *V* > 0, which is the range where the NDR effect is observed, the total ionic conductance *G* of the pore scaled to the maximum surface conductance *G*_0_ is
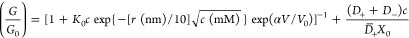
2where *D̅*_+_ < *D*_+_ is the cation surface diffusion coefficient^25^ and *D*_+_ and *D*_–_ are the cation and anion diffusion coefficients of the pore center bulk solution, respectively. The first term of eq 2 gives the surface conduction of the free cations in the vicinity of the pore charges of concentration *X* (eq 1).^25^ The second term corresponds to the bulk conduction of the cations and anions at the pore center region. In this rather artificial two-region model, we assume that the above dimensionless contributions to the total conductance occur over pore regions of similar cross-section area.^25^ This assumption should be reasonable here because it is a necessary condition for the NDR phenomena to be observed, as suggested by the effect of increasing the pore radius in Figure 6a. In fact, the uncertainties concerning the surface and pore center areas available for conduction,^25^ together with the non-cylindrical pore geometry,^14,20^ make it difficult to estimate absolute values of total conductances and currents. Instead, we will consider a dimensionless *I*–*V* curve written in terms of reference values for the current *I*_0_, potential *V*_0_, and resistance *R*_0_ as
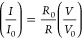
3For *I*_0_ = 1 nA and *V*_0_ = 26 mV, *R*_0_ = *V*_0_/*I*_0_ = 26 MΩ. The nanopore resistance *R* can be separated into the different conductance regimes of the *I*–*V* curve of Figure 1 as a function of the applied time (*t*)-dependent voltage *V*
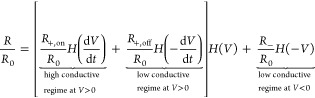
4where *H*(*x*) is the Heaviside function of argument *x*, defined as *H*(*x*) = 1 for *x* > 0 and *H*(*x*) = 0 otherwise, and *R*_–_ is the experimental resistance for the rectified current obtained at *V* < 0. From eq 2 for *V* > 0, the *on* and *off* resistances of eq 4 can be defined as
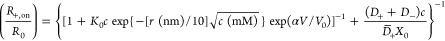
5
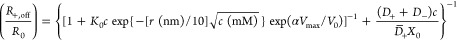
6where *V*_max_ is the experimental maximum value (signal amplitude) of the applied potential.

It is in order now to estimate plausible values for the system parameters. From the association constant *K*_0_, we can define the reference concentration *c*_0_ = 1/*K*_0_ that should be in the range 10–100 mM for the NDR effects observed here. As to the effective radius of the pore tip zone, it should be of the order of 10 nm (Figure 6a). The dimensionless electrical distance, however, is rather uncertain and can tentatively be assumed in the range^26^ α = 0.1–0.2 for reasonable voltage drops α*V* at the pore tip zone.^14^ For the volume pore charge concentration, a maximum value *X*_0_ = 1000 mM should be introduced.^14^ As to the surface diffusion coefficient, it can be decreased by a factor 10 compared with the bulk solution values.^24,25^ Thus, if we assume *D*_+_ = *D*_–_ to estimate the bulk conductance term of eq 2, we obtain 2*D*_+_*c*/(D*®*_+_*X*_0_) = *D*_r_(2*c*/*X*_0_) = 0.02 for *c* = 1 mM, where we have used *D*_r_ = (*D*_+_/*D̅*_+_) = 10 for this salt-dependent parameter.

**Figure 10:**
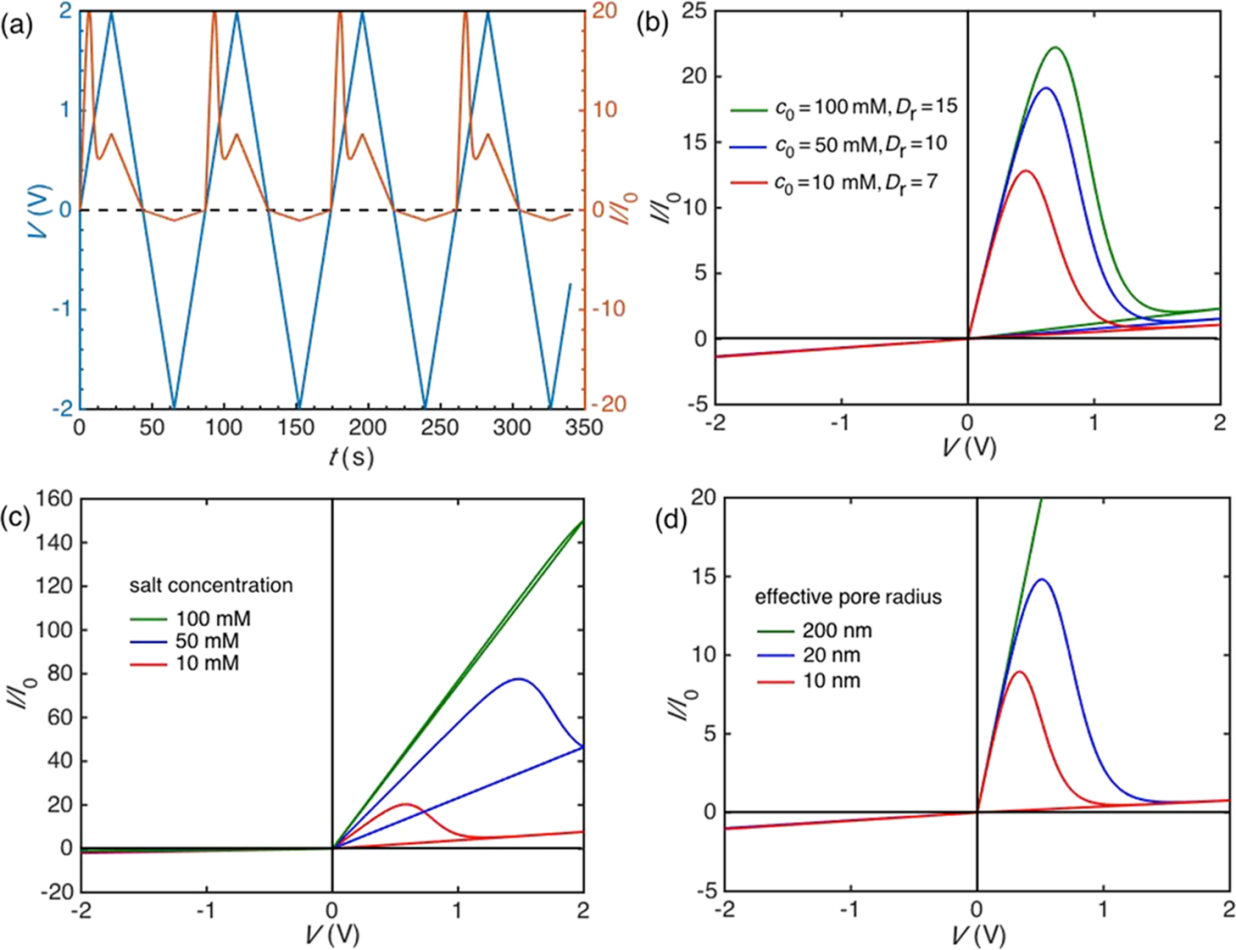
(a) Applied voltage *V*(*t*) and dimensionless current *I*(*t*)/*I*_0_ vs time *t* obtained for *c*_0_ = 1/*K*_0_ = 20 mM and *D*_r_ = 10 at the salt concentration *c* = 10 mM. (b) *I*(*t*)/*I*_0_–*V* curves for different cations characterized by distinct values of *c*_0_ = 1/*K*_0_ and *D*_r_ in eq 2 at *c* = 2 mM. (c) *I*(*t*)/*I*_0_–*V* curves obtained at different concentrations *c* for *c*_0_ = 20 mM and *D*_r_ = 10. (d) *I*(*t*)/*I*_0_–*V* curves obtained with different pore radii *r* at *c* = 1 mM for *c*_0_ = 20 mM and *D*_r_ = 10. In the figures, α = 0.2 in eq 2, *R*_–_ = 2 GΩ in Equation 4, and *r* = 20 nm (except for case (d)).

Figure 10a–d suggests that eqs 3–6 could allow for a qualitative description of the current–time (Figure 10a) and current–voltage curves for the cases of: (i) different cations characterized by the salt-dependent association constant *K*_0_ (reference concentration *c*_0_ = 1/*K*_0_) and *D*_r_ (Figure 10b), (ii) different salt concentrations *c* (Figure 10c), and (iii) different pore radii *r* (Figure 10d). Compare, in particular, the observed trends of Figures 1, 2d, 4a, and 6a with the theoretical curves of Figure 10b–d, respectively, which have been obtained for plausible values of the system parameters.

Note also that Figure 10c,d predict that no threshold voltage should be observed for the cases of high concentration because of the high Debye screening of the pore surface charges, and wide pore tips because of the low pore charge concentration, as observed experimentally. These facts may justify the result of having a threshold voltage that increases with the availability of ionic carriers. Note that, in the model, the increase in the Debye screening of the pore charges that occurs at high salt concentrations leads to a decrease of the effective electrostatic barrier at the pore tip seen by the F^–^ ions. Thus, the fluoride ions can proceed through the pore with no accumulation at the tip zone in this case, which could justify the absence of the NDR effect.

In summary, this qualitative model interprets the resistance switching in terms of the progressive accumulation (*V* > 0) and depletion (*V* < 0) of the F^–^ ions at the pore tip^14^ and their subsequent effect on the interaction of the cations with the negative pore surface. At *V* > 0, the fluoride ions interacting strongly with the water molecules are progressively accumulated at the effective barrier created by the negative pore tip charges. At high enough voltages, the F^–^ accumulation and decrease of free water molecules enhances the cation interaction with the pore surface charges at the tip modulating the current,^14^ thus decreasing the surface conductance. At *V* < 0, on the contrary, the F^–^ ions are progressively depleted from the tip.^14^ Thus, the pore recovers the high conductance state at the beginning of next cycle, from *V* = 0 up to positive voltages close to the threshold where F^–^ accumulation becomes significant again. The experimental fact that the positively charged PEI-modified pore does not show any NDR phenomena (Figure 6c) supports our interpretation of the negatively charged pore tip as a kinetic barrier for F^–^. Because the PEI-modified pore is positively rather than negatively charged, no kinetic barrier should exist at the tip in this case.

The limited understanding of the observed phenomena, together with the limitations of the continuum mean field description at the pore tip,^27,28^ have resulted in the above phenomenological approach. Future models could consider also the effect of the interactions between the imide rings, the fluoride ion, and the cation as well as the resulting microscopic charge correlation phenomena.

## Conclusions

We have given a complete experimental and theoretical characterization of the NDR phenomena observed in conical nanopores at low fluoride salt concentrations under a wide range of operating conditions. The experimental data obtained with single pore and multipore samples concern different pore radii, charge concentrations, scan rates, salt concentrations, solvents, and cations. Under the NDR conditions, small voltage changes around 1 V can amplify weak electrical perturbations, an effect potentially useful for nanofluidic sensing and actuating. The theoretical approach is based on a two-region conductance model where the mobile cations in the vicinity of the negative pore charges are responsible for the surface conductance while bulk solution conductances are assumed in the pore center region. The model explains the conductance switching in terms of the progressive accumulation/depletion of the fluoride ions at the pore tip zone and the resulting effects on the interaction of the cation with the surface charges. The NDR phenomena reported should have both fundamental and practical interest.
